# The impact of an integrated early palliative care telehealth intervention on the quality of life of heart failure patients: a randomized controlled feasibility study

**DOI:** 10.1186/s12904-024-01348-z

**Published:** 2024-01-23

**Authors:** Arvin Mirshahi, Marie Bakitas, Meysam Khoshavi, Ali Khanipour-Kencha, Seyed Mohammad Riahi, Rachel Wells, J. Nicholas Odom, Shahrzad Ghiyasvandian, Masoumeh Zakerimoghadam

**Affiliations:** 1 Students’ Scientific Research Center, Department of Medical-Surgical Nursing, School of Nursing and Midwifery, Tehran University of Medical Sciences, Tehran, Iran; 2 Center for Palliative and Supportive Care, University of Alabama at Birmingham, Birmingham, AL USA; 3 School of Nursing, and Department of Medicine, Division of Gerontology, Geriatrics, and Palliative Care, University of Alabama at Birmingham, Birmingham, AL USA; 4 Department of Cardiology, School of Medicine, Imam Khomeini Hospital, Tehran University of Medical Sciences, Tehran, Iran; 5 Department of Community Medicine, School of Medicine, Cardiovascular Diseases Research Center, Birjand University of Medical Sciences, Birjand, Iran; 6 School of Nursing, University of Alabama at Birmingham, Birmingham, AL USA; 7 School of Nursing, University of Alabama at Birmingham (UAB) and UAB Center for Palliative and Supportive Care, Birmingham, AL USA; 8 Department of Medical-Surgical Nursing, School of Nursing and Midwifery, Tehran University of Medical Sciences, Nosrat St., Tohid Sq, Tehran, Post Code: 14197-33171 Iran

**Keywords:** Telehealth, Palliative care, Heart failure, Feasibility study, Quality of life, Iran

## Abstract

**Background:**

While palliative care for patients with heart failure has gained global attention, in Iran most palliative care interventions have focused only on cancer patients. The purpose of this study is to determine the feasibility and acceptability of a telehealth palliative care intervention to improve the quality of life in patients with heart failure in Iran.

**Methods:**

This single-site, pilot randomized controlled trial of a telehealth palliative care intervention versus usual care was conducted on patients with New York Heart Association class II/III heart failure recruited from a heart failure clinic in Iran. Under the supervision of a nurse interventionist, intervention participants received 6 weekly educational webinars and concurrent WhatsApp® group activities, with 6 weeks of follow-up. Feasibility was assessed by measuring recruitment, attrition, and questionnaire completion rates; acceptability was assessed via telephone interviews asking about satisfaction and attitudes. Secondary outcomes measured at baseline and 6 weeks included quality of life (PKCCQ and FACIT-Pal-14), anxiety and depression (HADS), and emergency department visits.

**Results:**

We recruited and randomized 50 patients (mean age 47.5 years, 60% men). Among those approached for consent, 66% of patients agreed to participate and total study attrition was 10%. Also 68% of patients successfully completed at least 4 out of the 6 webinar sessions. Acceptability: 78% of patient participants expressed willingness to participate in the present study again or recommend other patients to participate. There was a trend towards improvement in anxiety and depression scores in the intervention group though the study was not powered to detect a statistical difference.

**Conclusion:**

This nurse-led, early telehealth-palliative care intervention demonstrated evidence of feasibility, acceptability, and potential improvement on quality of life in patients with heart failure in Iran.

**Trial registration:**

The study was registered at the Iranian Registry of Clinical Trials (IRCT) at 14 November, 2021, and can be found on the Iranian Registry of Clinical Trials Platform.

IRCT registration number: IRCT20100725004443N29.

## Background

Heart failure is a common chronic condition which globally, the number of heart failure cases has nearly doubled to more than 64 million from 1990 to 2017, and this number is expected to continue to increase over the next few decades [1]. In Iran, the prevalence of heart failure is estimated to be about 8% [2], and the one-year mortality rate for these patients is remarkably high at 32% [3]. This condition poses various challenges such as high rehospitalization and in-hospital mortality rates [4]. Despite advances in medical management, patients with heart failure often experience symptoms such as dyspnea, fatigue, and pain, which can significantly impact their quality of life [5]. Early integration of palliative care has emerged as a potential strategy to address these symptoms and improve outcomes in patients with heart failure [6, 7].

Iran currently lacks a well-defined and specific framework for providing palliative and supportive care [8]. This care is provided, with cancer patients being the primary recipients. Unfortunately, there is a lack of a care for incurable patients, leading to inadequate facilities, a shortage of trained healthcare professionals, and a lack of proper organizational structures to support these services [9, 10]. Although access to palliative care services in Iran is limited [11, 12], the situation has been further exacerbated by the COVID-19 pandemic, which has underscored the urgent need for alternative methods of delivering care [13, 14].

Telehealth has emerged as a potential solution to address the challenges of providing palliative care [15, 16]. Telehealth is a valuable healthcare solution with several advantages. First, it offers convenience to patients by allowing them to access medical services from home. Second, during infectious disease outbreaks like COVID-19, telehealth ensures safety by reducing the risk of exposure in healthcare settings [17]. Third, virtual consultations also strengthen family connections as loved ones can participate in the patient's healthcare journey [18]. Fourth, telehealth can lead to reduced health service costs compared to in-person visits [19]. Finally, it plays a crucial role in preserving physical health service capacity for urgent cases and is particularly beneficial for populations in remote areas with limited access to specialized medical centers [20]. In Iran, the shortage of adequately trained healthcare professionals in palliative care and the absence of suitable organizational structures to support such services [9, 10] make telehealth palliative care a promising approach to alleviate patient issues and enhance their quality of life.

Telehealth has been shown to be effective in delivering palliative care services to patients with heart failure, as it allows for remote monitoring, symptom management, and communication between patients and healthcare providers [21]. Considering the lack of prior palliative care telehealth interventions in Iran, it is imperative to conduct a comprehensive assessment of the feasibility and effectiveness of telehealth-based palliative care interventions in this region.

Consequently, we conducted a randomized controlled feasibility study to evaluate the integration of an early nurse-led, twelve-week telehealth-based palliative care intervention consisting of weekly webinar sessions and WhatsApp group activities for Iranian patients with New York Hospital Association (NYHA) Class II/III heart failure. This intervention is based on Wagner’s Chronic Illness Care (CIC) model [22, 23], and the foundation of the ENABLE CHF-PC intervention [21, 24–28], incorporating both case management and educational strategies to encourage patient activation, self-management, and empowerment. Additionally, peer education has been included as part of this approach and a follow-up period to ensure comprehensive implementation of the program. By evaluating intervention feasibility and acceptability and its potential impact on patient outcomes such as quality of life, mood status and emergency department visits, this study provides valuable insights into the potential of telehealth to enhance access to palliative care services in Iran and inform future research and clinical practice, ultimately leading to improved heart failure patient outcomes.

## Methods

### Study design

Following the Consolidated Standards of Reporting Trials (CONSORT) [29] this was a single-site, pilot randomized controlled trial (intervention vs. usual care) among adults with a diagnosis of New York Heart Association (NYHA) class II or III heart failure (HF) or American College of Cardiology (ACC) stage B or C HF. The study protocol has been published elsewhere [30]. The human participants' protocol and data safety monitoring plan were approved by the Tehran University of Medical Sciences Institutional Review Boards (IR.TUMS.FNM.REC.1400.071). Patients provided written informed consent that was prepared according to the Declaration of Helsinki [31].

### Participants

Study participants were identified and recruited from Imam Khomeini Hospital Complex (IKHC) HF clinic, affiliated with Tehran University of Medical Sciences, by a trained nurse interventionist using screening algorithms and under the supervision of an HF clinician (from January 20, 2022, to July 1, 2022). Eligibility criteria were: (1) age 18–65 years, (2) clinician-determined NYHA class II/III or ACC/AHA stage B/C HF, (3) ability to read and write Persian language, (4) access to and ability to use personally-owned smartphones, and (5) ability to use WhatsApp® messenger. The exclusion criteria included: (1) having cognitive disorders documented in the medical record, (2) having uncorrectable vision and hearing disorders, (3) existence of a comorbid disease that requires specialized palliative care such as cancer, and (4) end-stage or exacerbation HF.

### Intervention

**Fig. 1:**
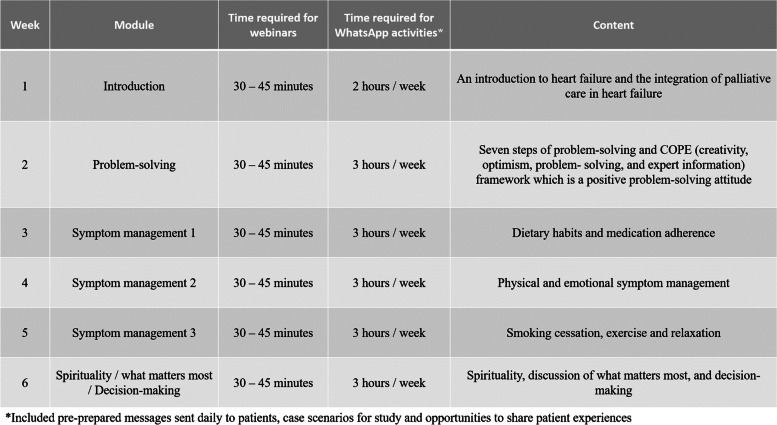
Descriptions of the intervention

The intervention has been described in detail elsewhere [30]. Briefly, a nurse interventionist conducted a comprehensive six-week virtual program for patients in the intervention group. The program consisted of weekly webinars that covered topics based on the ENABLE CHF-PC (Educate, Nurture, Advise, Before Life Ends Comprehensive Heartcare for Patients and Caregivers) program [21], American Heart Association and The European Society of Cardiology guidelines [32–34]. To supplement the webinars, the intervention group received a six-chapter booklet titled “Palliative care in patients with heart failure” during an in-person visit by the HF specialist and the nurse interventionist before starting the program. In brief, Chapter 1 introduces heart failure and palliative care; Chapter 2 discusses COPE (creativity, optimism, problem-solving, and expert information), a positive problem-solving attitude and seven problem solving steps. Chapters 3, 4 and 5 address self-care and symptom management and includes dietary habits, medication adherence, physical and psychological symptom management, smoking cessation, exercise, and relaxation. Chapter 6 comprises spirituality, discussion of what matters most, and decision-making. The webinars were held on an Iranian platform called SkyRoom® and lasted for 30–45 min. After each session, patients could share their thoughts and questions about the topic discussed on the webinar. Patients also joined WhatsApp® groups where they received standardized content corresponding to each week's webinar topic. Furthermore, the nurse interventionist sent case scenarios to the WhatsApp® groups and encouraged patients to discuss and comment on them synchronously. This part of the intervention aimed to engage patients with case scenarios and empower them to make appropriate decisions when facing similar situations. Additionally, it focused on peer education to improve patients' understanding of the webinar content and learn from other patients' experiences. Lastly, patients were followed up for six weeks after completing the virtual program (until November 30, 2022). Throughout the follow-up period, the nurse interventionist remained available through different communication channels, including text messages, phone calls, and WhatsApp messenger, to offer any required support, assistance, and address any inquiries, concerns, or emergencies that the patients might have had. This ongoing support and availability of the nurse interventionist during the follow-up phase ensured that patients received the necessary care and guidance beyond the virtual program's completion. Patients randomized to the usual care group continued to receive the same outpatient management as prior to enrolling in the study. In the IKHC cardiology clinic, routine HF care consisted of in-person visits performed by HF specialists and brief instructions about dietary habits, physical activity, taking medications as prescribed, and weight monitoring. For ethical considerations, the digital content about HF self-care was sent to control group patients via WhatsApp® at the end of the study. A schematic representation of the intervention is shown in (Fig. 1).

### Data collection

#### Primary end point: feasibility and acceptability

Feasibility was measured by determining the recruitment questionnaire and intervention sessions completion rates. Adequate feasibility metrics for this study included: 80% of interested individuals agreeing to screening; 60% of those eligible enrolling into the trial; baseline assessment completion (> 80%), attrition rate (< 20%) and completion of at least 4 out of 6 webinar sessions and good engagement in WhatsApp group activities as observed by the nurse interventionist. The criteria for successful engagement included active participation in weekly WhatsApp group discussions, effective collaboration with fellow patients, and providing feedback to the nurse interventionist regarding the questions posed during the intervention process.

The acceptability of the study was measured by a telephone-based interview where participants rated their adherence to the intervention (10-point Likert scale), satisfaction with the webinar sessions (5-point Likert scale), satisfaction with the WhatsApp group activities (5-point Likert scale) and if they would do the study again or recommend it to others (yes/no). The study procedures and intervention were considered “acceptable” as evidenced by their adherence to the intervention (≥ 7), satisfaction with the interventions (webinars and WhatsApp group activities) (≥ 4), and if they would do the study again or recommend it to others (≥ 75%). We encouraged patients to share their thoughts on the intervention. They comments were transcribed verbatim and analysed according Graneheim and Lundman’s method [35] by MZ.

#### Secondary end points: quality of life, mood status and number of emergency department visits

Secondary outcomes were quality of life measured by: (1) The Persian version of the Kansas City Cardiomyopathy Questionnaire (PKCCQ) [36] (score range, 0-100; higher scores indicate better perceived health status; clinical summary scores ≥ 50 are considered “fairly good” quality of life [37, 38] and a change of 5 points is considered a clinically important difference [39]), and (2) the 14-item Functional Assessment of Chronic Illness Therapy–Palliative-14 questionnaire (FACIT PAL-14) [40] (score range, 0-56; higher scores indicate better quality of life [41]). Mood was measured by the 14-item Hospital Anxiety and Depression Scale (HADS) [42], where 7 items measure anxiety symptoms and 7 items measure depressive symptoms (subscale ranges, 0-21; scores > 8 indicate clinically high symptoms [43]; and a change of 1.7 is considered a clinically important difference [44]). Also, the number of emergency department visits was obtained by a blinded outcome assessor. In the case of admission to other healthcare settings except for IKHC, data was collected according to the participants’ self-reports. If the participant was admitted to IKCH, the hospital information system (HIS) confirmed the data following the participant’s self-report. The number of visits was reported within the 12 weeks after enrolment.

A trained assessor who was blinded collected participants’ baseline survey including PKCCQ, FACIT Pal-14, and HADS, during the in-person IKCH HF clinic visit and week 12 using paper-and-pencil questionnaire. The main outcome of the study (feasibility and acceptability) was measured by a trained interviewer.

### Sample size

For the pilot nature of this study, the sample size was determined by the precision of estimates, measured by 95% confidence interval (CI) [45]. A sample of 50 participants was deemed sufficient to assess feasibility and acceptability of the intervention and the clinical trial procedures [46]. For example we expected 60% enrolment rate, 80% completion rate, and 20% attrition rate, the 95%CIs for observed rates with a sample size of 50 would be 49%-71%, 69%-91%, and 9%-31%, respectively. It is important to acknowledge that the fundamental essence of this study is exploratory in nature, not confirmatory. As a result, the primary emphasis for secondary outcomes is not on statistical power, as the study avoids making any inferences. Instead, it employs descriptive statistics that are well-suited for examining pilot data for the precision of estimated location (mean) and variability. Assuming 10% attrition, the 95%CI of estimated effect would be within ± 0.5 standard deviation. However, the sample size of 50 (N2/N1 = 1) would achieve 80% power to detect a large effect size (Cohen’s d = 0.9) if such effect size could exist, using a two sample t-test at the significance level of 0.05, assuming 10% attrition.

### Randomization and blinding

Before completing baseline questionnaires, patients were randomized by project manager (M.Z) to either the intervention or the usual care group using a stratified block randomization method. Patients were randomized by 1-to-1 assignment, with a block size of 4. Randomization was stratified by gender and NYHA classification of heart failure (Class II or III of HF). The nurse interventionist notified participants about their allocation status. Due to the nature of the study, participants and the nurse interventionist were aware of allocation to the intervention group. Participants were asked not to discuss their study activities with the outcome assessor. The outcome assessor and statistician were blinded to the study groups.

### Statistical analysis

Mean and standard deviation (SD) were used to describe numerical variables. As for categorical variables, frequency tables were utilized as a percentage. The groups’ participation rate, response rate, and attrition rate were reported as a percentage and a 95% confidence interval (95%CI) for measuring the intervention’s feasibility and acceptability. The study was not powered to detect a statistically significant difference in the secondary outcome measures. For analyzing the secondary outcomes, we placed more emphasis on descriptive statistics that can be used to (1) obtain a tentative estimate of efficacy to determine whether the signal is large enough to proceed with a larger scale trial of effectiveness; and (2) determine SD of outcome measures to facilitate sample size planning of subsequent larger trial. In addition, inferential statistics were also conducted. The Shapiro–Wilk test was used to check the normality of the data distribution. The independent T- test compared numerical variables and Chi-square test examined nominal variables in the two groups at the baseline. The paired T-test was used to evaluate the effectiveness of the intervention within groups. Analysis of covariance (ANCOVA) was applied to adjust confounding variables. Data analysis were conducted using SPSS software (Version 22). All tests were two tailed and statistical significances was defined as *p* < 0.05.

## Results

### Feasibility/acceptability: Intervention, measure completion and satisfaction

**Fig. 2:**
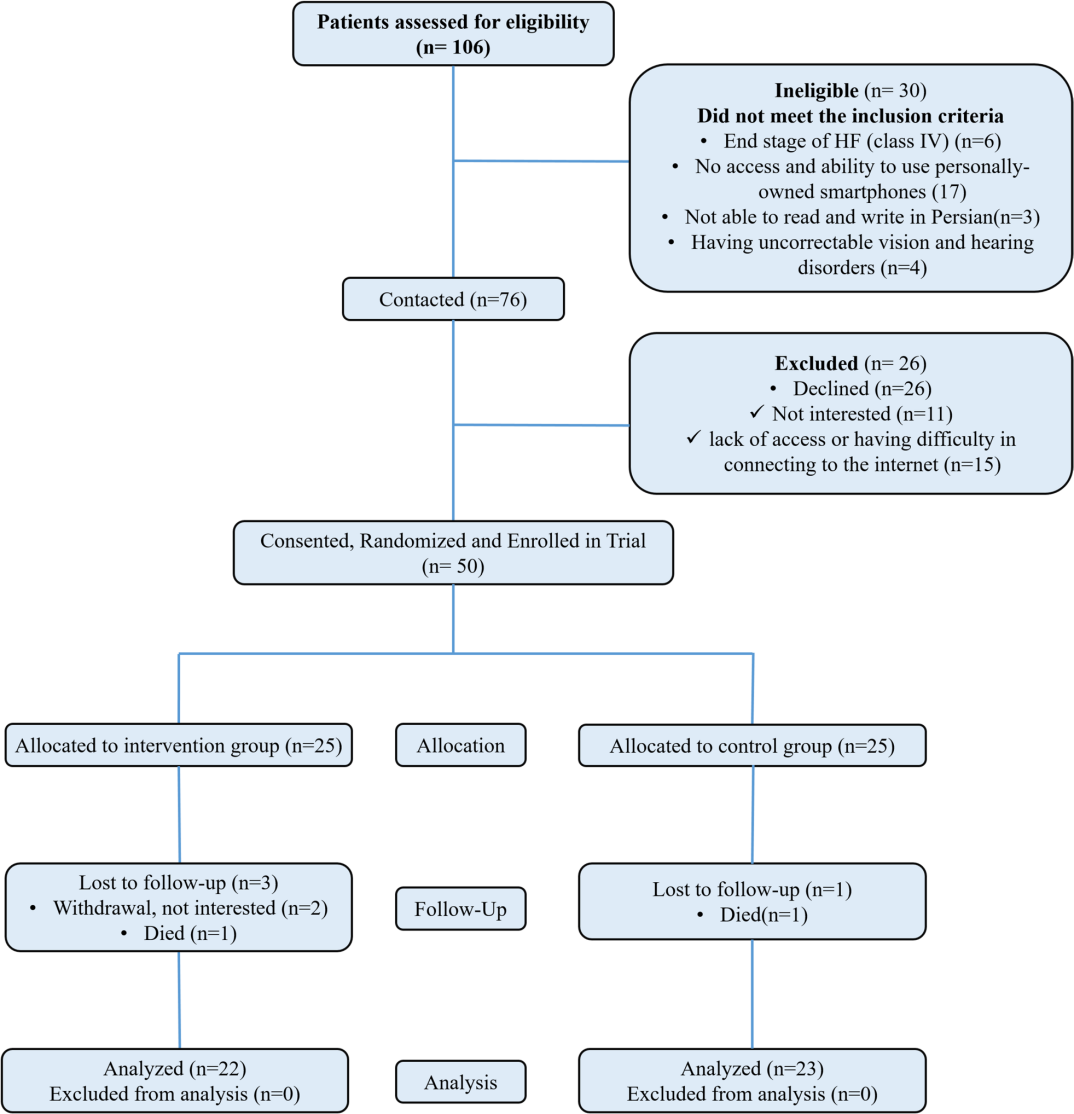
CONSORT diagram: Patients recruitment, treatment, and analysis

A total of 106 patients were evaluated to determine eligibility, as shown in Fig. 2. Of the 76 eligible patients (71% patients assessed for eligibility), 26 patients declined to participate due to a lack of interest in the intervention. The remaining 50 patients (66% acceptance rate) enrolled in the study were randomized to receive the early telehealth-palliative care intervention (*n* = 25) or usual care (*n* = 25). Baseline characteristics were balanced between groups (Table 1). Overall, 50 patients were a mean age of 47.5 years, most were male (60%, *n* = 30), married or living with a partner (80%, *n* = 40), were smoking (40%, *n* = 20) self-employed or housekeeper (66%, *n* = 33), had high school or general education diploma (72%, *n* = 36), had insurance (96%, *n* = 48), had fair to poor income (82%, *n* = 41) and were urban (78%, *n* = 39). Clinically, 50% patients were NYHA Class III with a mean ejection fraction of 21.4. Attrition rate was due to withdrawal 3 (6%) and death 2 (4%).

**Table 1:** Baseline Characteristics of Participants

**Characteristic**	**Participants, No. (%)**		***P* value**
	Intervention (*n* = 25)	Usual care (*n* = 25)	
**Age, mean (SD)**	44.68 (11.90)	50.32 (10.19)	0.08
**Sex**			
**Male**	13 (52)	17 (68)	0.24
**Female**	12 (48)	8 (32)	
**Marital Status**			
**Single**	7 (28)	1 (4)	0.52
**Married**	18 (72)	22 (88)	
**Divorced**	0 (0)	2 (8)	
**BMI**	25.97 (5.28)	25.94 (5.13)	0.98
**Education, frequency (%)**			
**Primary education**	12 (48)	11 (44)	0.59
**Diploma**	6 (24)	10 (40)	
**Higher education**	7 (28)	4 (16)	
**Job Status**			
**Employed**	4 (16)	3 (12)	0.50
**Self-employed**	8 (32)	12 (48)	
**Housekeeper**	9 (36)	4 (16)	
**Unemployed**	1 (4)	2 (8)	
**Retired**	3 (12)	4 (16)	
**Smoking**			
**Yes**	9 (36)	11 (44)	0.56
**No**	16 (64)	14 (56)	
**Residency**			
**Urban**	19 (76)	20 (80)	0.73
**Rural**	6 (24)	5 (20)	
**Income Status**			
**Enough**	4 (16)	5 (20)	0.21
**Fairly enough**	6 (24)	11 (44)	
**Not enough**	15 (60)	9 (36)	
**Insurance**			
**Yes**	23 (92)	25 (100)	0.15
**No**	2 (8)	0 (0)	
**Ejection fraction at enrollment, Mean (SD), %**	20.4 (8.4)	22.4 (8.79)	0.82
**Patient-reported Outcomes, Mean (SD)**			
**Primary (Quality of Life)**			
**PKCCQ clinical summary**	49.53 (13.2)	50.85 (9.60)	0.68
**FACIT Pal-14**	32.4 (8.98)	34.44 (7.69)	0.39
**Secondary (Mood Status)**			
**HADS**			
**Anxiety**	8.13 (3.28)	8.08 (3.54)	0.93
**Depression**	7.09 (3.03)	7.21 (3.06)	0.89

Questionnaire completion rates were high at the initial time point, demonstrating that the instruments and delivery methods used were acceptable. However, due to attrition, only 45 (90%) patients filled out the questionnaire at both time-points. Regarding the completion of intervention sessions, 15 (68%) patients attended at least 4 out of 6 webinar sessions. From the perspective of the nurse interventionist, the engagement of the patients in WhatsApp group activities were acceptable. We successfully recruited our target population of people with heart failure. Regarding acceptability, after a telephone-based interview conducted by a trained interviewer, the mean score of patient’s adherence to the intervention was reported 7 ± 1.02 [30 patients (66.6%) with satisfaction score ≥ 7]. Regarding the satisfaction with the intervention, which categorized to satisfaction with webinar sessions and WhatsApp group activities, the mean scores were reported 3.71 ± 0.94 [29 patients (64.4%) with satisfaction score ≥ 4] and 4.02 ± 0.81 [33 patients (73.3%) with satisfaction score ≥ 4], respectively. In relation to recommending others to participate in the intervention, based on their satisfaction with the study, 35 patients (77.7%) have expressed their willingness to participate in the present study again or recommend other patients to take part in the intervention.

### Patients perspectives on acceptability of the intervention

Among the 45 patients who successfully completed the study and participated in interviews, 14 patients conveyed their perspectives, whereas the remaining individuals did not provide any feedback regarding their viewpoints. Tables 2 and 3 highlights several patients comments regarding acceptability of the webinar sessions and WhatsApp group activities.

**Table 2:** Patient’s Perspectives on Acceptability of the Intervention

	**Patient’s Characteristics (perspectives)**	**Patient’s Comments**
**Patient’s perspectives about Webinar sessions**	Patient #13; 61-year-old; Female (Positive attitude)	*“For me, as a 61-year-old woman who doesn’t have any experience working with smartphone’s applications at all, working with this platform and having our weekly sessions was very easy. I just tried once to learn from my son how to work with it. The voice and the video of the lecturer was very clear and we could collaborate with him *via* activating our microphones or just by sending messages through the text box of the platform*.”
	Patient #18; 43-year-old; Male (Positive attitude)	*“The platform was very user-friendly and the most important thing that made it better was that for using it, you didn’t need a high-speed internet. Because as a teacher, I used a lot of platforms in the Quarantine period during COVID-19 for having my classes and approximately, due to the Low internet speed in Iran, we had an internet issue with most of the platforms, like kicking out of the room because of bad connection or not having a good and clear voice and video. I didn’t have this problem with this platform as much as others.”*
	Patient #2; 53-year-old; Female (Negative attitude)	“*Personally, I was Ok with some sessions of the webinars but, I don’t think that it would be good way to talk about some issues like death and decision-making in a webinar session that there are 10 other people are listening! I rather prefer to talk about these subjects face to face and privately.”*
	Patient #8; 42-year-old; Male (Positive attitude)	“*The whole concept of the webinar sessions was good and it was informative for me, the time of the sessions wasn’t too much so it was not time-consuming. We could easily schedule for a 30-min class in a week! But one thing that I thought it can make the program better, is that you can record the sessions and send the video of it for us so we can watch the video whenever we came across a question. Of course still I think a face to face meeting would be better for having this kind of programs!”*
	**Patients Characteristics**	**Patients Comments**
**Patient’s perspectives about WhatsApp group Activities**	Patient #3; 39-year-old; Male (Positive attitude)	*“I think this part of the program was great! As a person with a 5-month history of heart failure, I really learned very much from other patients who were struggling with heart problem for several years.”*
	Patient #22; 44-year-old; Female (Positive attitude)	*“The tasks that was given to us, was one of the main positive points of this program. It was very interesting that we had to respond to the case scenarios given by the facilitator in the group according to our knowledge, training, and also experiences expressed by the patients. It is very important and valuable to me to be able to take care of myself if I find myself in a similar situation."*
	Patient #1; 43-year-old; Male (Negative attitude)	*“Although the program was useful, I think I didn’t really like the way of communication in the WhatsApp groups, I wasn’t very comfortable expressing my attitude in the group where other patients were there*

**Table 3:** Outcomes Score Change from Baseline to Week 12 (Intervention vs Usual Care)

Outcomes	Intervention Group (*n* = 22)	Usual Care Group (*n* = 23)	Difference Between Change Scores (95% CI)	*P* value
	Mean Observed Change From Baseline (SD)			
PKCCQ clinical summary	0.69 (0.47)**	-0.94 (1.31)**	-1.54 (-2.14 to -0.94)	** < 0.001**
FACIT Pal-14	2 (2.04)**	-0.69 (2.93)	-2.69 (-1.16 to 4.22)	**0.001**
HADS-Anxiety	-1.10 (1.54)**	-0.78 (1.41)*	0.31 (1.20 to -0.57)	0.45
HADS-Depression	-0.59 (1.18)*	-0.30 (1.86)	0.28 (1.23 to -0.65)	0.54

^*^P value < 0.05

^**^P value < 0.01

### Patient reported outcome measures

As a feasibility study, it was not designed or powered to detect changes, we examined whether there was a signal for intervention effects on Quality of life, mood status and Emergencey Department (ED) visits (Tables 3 and 4).

**Table 4:** Analysis of covariance (ANCOVA) by adjusting the relevant variables according to the baseline score

Outcomes		Raw mean		Adjusted mean^a^		*P* Value	*R*^2^
		Control Mean (SD)	Intervention Mean (SD)	Control Mean (SD)	Intervention Mean (SD)		
Quality of life	PKCCQ	49.10 (8.86)	51.44 (12.14)	49.20 (9.07)	51.62 (11.90)	**0 < 0.001**	0.99
	FACIT Pal-14	33.26 (6.92)	35.13 (7.62)	32.90 (2.41)	35.50 (2.41)	**0.001**	0.89
Mood status	Depression	6.91 (2.55)	6.50 (2.42)	6.87 (1.26)	6.54 (1.28)	0.713	0.73
	Anxiety	7.30 (3.13)	7.04 (2.80)	7.32 (1.29)	7.02 (1.32)	0.446	0.81

^a^The indicators have been adjusted according to the variable baseline score

#### Quality of life

Data analysis revealed statistically significant improvements in quality of life (PKCCQ and FACIT PAL-14) from baseline to week 12. At week 12, compared to baseline, there was a significant change in mean PKCCQ scores of intervention group than control group (mean difference, -1.54 [95% CI, -2.14 to -0.94, *p* < 0.001]). FACIT PAL-14 scores, showed a significant change in mean scores from baseline to week 12 of intervention vs control group (mean difference, -2.69 [95% CI, -1.16 to -4.22, *p* = 0.001]).

#### Mood status

The HADS questionnaire showed that there was no significant improvement in anxiety and depression of patients between baseline and week 12. There was no significant change in mean HADS anxiety subscale (mean difference, 0.31 [95% CI, 1.20 to -0.57, *p* = 0.45] and depression (mean difference, 0.28 [95% CI, 1.23 to -0.65, *p* = 0.54].

#### ED visits

There were not significant differences in the number of patient’s ED visits between intervention (1.13 ± 1.12) vs control group (1.4 ± 1.32, 95% CI, -0.57 to 0.91, *p* = 0.45] within 12 weeks after enrolment.

## Discussion

Given the lack of culturally based, accessible early telehealth palliative care intervention for patients with HF, the purpose of this randomized controlled feasibility trial was to determine the feasibility and acceptability of implementing an early telehealth palliative care intervention for heart failure patients in Iran. We demonstrated that the early telehealth palliative care intervention was feasible and acceptable to Iranian heart failure patients. There was also improvement in quality-of -life outcomes, however the study was not powered to examine effect.

### Key findings of the study

There were numerous key lessons learned that can inform future intervention development.

First, most patients who were approached for study participation did not have accurate information about palliative care and what it entails; however, after receiving a recommendation by their physician and study information most agreed to participate. Similar to the findings of others [12, 47, 48], patients in our study believed that palliative care was equivalent to end-of-life care received in their last six month of live and was only available to cancer patients who had no hope of being cured. The awareness of the Iranian community and the general public about palliative care is very low and many patients will refuse such care due to their lack of knowledge. This underscores the importance of widespread education about palliative care in Iran. This important point was also evident in a study conducted to investigate palliative care knowledge among Iranian cancer patients [12] which also demonstrated that most cancer patients had a poor level of knowledge and had significant misconceptions about palliative care, highlighting the importance of providing palliative care education. As long as patients do not have accurate and reliable information about palliative care, they will likely still refuse to receive it even if it is widely available.

Second, internet-based interventions are thought to be commonly associated with poor adherence and high attrition rates [49]. Providing palliative care to patients in this study was challenging due to the remote and internet-based nature of the intervention. Limited access to the internet in some parts of the country, especially rural areas, was one of the challenges encountered. The study was conducted at the heart failure clinic affiliated with Tehran University of Medical Sciences, which is a highly regarded government-run medical center. Patients from all corners of the country, particularly those from underprivileged regions, seek treatment at this clinic. During the sampling process, 15 out of 26 individuals who declined to participate in the study cited lack of access and difficulty in connecting to the internet as their reasons. During the intervention, patients generally participated well in webinars and WhatsApp group activities. However, some patients reported occasional internet disconnections during the webinars, causing them to miss parts of the class content. These patients requested the instructor to repeat the material once they re-entered the class. Similar problems were also experienced by some patients during WhatsApp group activities, making it impossible for them to submit completed assignments easily. Such issues were more common among patients in rural areas, whereas the patients living in cities did not experience any internet-related problems.

This challenge highlights the difficulty of providing internet-based remote interventions in rural areas with low-speed internet, which could potentially reduce patients' satisfaction with receiving such interventions. In a qualitative study conducted by Dionne-Odom et al. [50] to adapt an initial palliative care intervention for family caregivers of individuals with advanced cancer residing in rural areas of the southern United States, study participants mentioned that due to poor access to the internet or the high cost of using it, they preferred not to receive care through the internet. Based on the results of this study and other studies [50, 51], telephone calls may be a better optionthe delivery of palliative care for rural patients. Despite these issues, palliative care can help overcome geographical barriers and allow patients to receive care from the comfort of their homes.

Third, cultural and ethical issues are a major challenge with group-based interventions. One of the main parts of palliative care is decision making and talking about things patients prefer to do before death [52]. Discussion of these topics is not easy for everyone, and in many countries, they are still considered taboo. According to a study [53], the end-of-life (EOL) wishes of half of the patients with chronic end-stage diseases, who have been living with these conditions for years, were not fulfilled. This indicates that many patients may be hospitalized, and even die, without having had the opportunity to express their EOL preferences [54, 55]. In this study, certain patients in the intervention group declined to express their thoughts and preferences regarding death and decision-making. This reluctance may be attributed to cultural factors, as there is a prevailing belief in Iranian culture that discussing death and dying with patients is unsuitable due to the potential for causing stress and anxiety and negatively affecting patient morale [56]. This aspect is evident in studies conducted to assess death anxiety among Iranian cardiovascular patients as well [57–59]. The findings of these studies reveal that patients with cardiovascular diseases experience a considerable level of anxiety, which may influence their reluctance to engage in discussions about death. The heightened anxiety related to their condition could contribute to their avoidance of such conversations. In our study, we faced some problems for encouraging some patients to talk about these issues. As noted in the interviews, one patient mentioned that he was not comfortable to express his opinion in social media group like WhatsApp, although, we also had some patients that were comfortable talking about these topics. A good option is giving a choice to patients as to whether they prefer to engage in group activities and share their ideas with others, or just sharing their preferences with the coach privately.

Another point that some patients highlighted in interviews was the permission they had to message or call the nurse interventionist whenever they needed help or advice. We encountered several emergency questions from patients, and after consulting with our HF specialist, some patients were referred to the hospital for more observation. Patients were very satisfied that they could call or message the nurse interventionist as normally they would need to call emergency services for their problem.

Many feasibility studies are not powered to detect significant differences in outcomes, rather the focus is on whether the intervention looks promising and can be evaluated [60]. Regarding the secondary outcomes, while this study was not designed for hypothesis testing, we noted improved quality of life from baseline to week 12. However, we did not observe a significant improvement in anxiety and depression and a reduction in ED visits.

We noticed that one of the main reasons for the substantial enhancement in patients' quality of life throughout the study period was a paradoxical change in PKCCQ clinical summary and FACIT-Pal-14 scores of patients in the intervention and usual care groups. Specifically, while patients in the intervention group experienced an improvement in their quality of life, some patients in the usual care group experienced a reduction in their quality of life scores. After conducting a detailed review of possible reasons, we noticed that some patients with significantly reduced quality of life, who received usual care were residents of cities located further away from Tehran, where the IKHC is located, as compared to the patients in the intervention group. This finding raised concerns about patients who face transportation barriers to accessing care. These barriers can impact the patient's ability to receive regular follow-up care and monitoring, which is critical for managing heart failure and preventing complications. The costs of traveling for medical appointments, as well as a lack of access to transportation, are commonly cited as major transportation barriers to healthcare [61]. Patients who are unable to access care and consultations due to transportation barriers may be at higher risk of hospitalization and other adverse outcomes [62]. This finding raises concerns about patients facing transportation barriers and highlights the importance of implementing telehealth interventions, such as the present study, to overcome geographical barriers and improve access to care for patients with heart failure. The present intervention not only helped maintain the quality of life of patients but also increased it, albeit by a small amount. This underscores the efficacy of telehealth interventions in improving patient outcomes and the need for further research into the potential benefits of such interventions.

There was a trend towards improvement in anxiety and depression scores in the intervention group though the study was not powered to detect a statistical difference. We anticipate conducting a fully powered trial so that we can properly test the intervention effect on these outcomes.

### Implications of the study

These findings demonstrate the feasibility and acceptability of early tele palliative care intervention for individuals with heart failure in a region where telehealth initiatives for this patient population are limited. This suggests a potential avenue for expanding palliative care services, particularly telehealth interventions, within the Iranian healthcare framework, shedding light on the viability of such programs for policymakers. Increasing the palliative care knowledge among both the general population and healthcare providers in Iran is of utmost importance and necessity. Currently, there is a lack of adequate understanding of palliative care, and addressing this knowledge gap is essential to improve the overall quality of care for patients with serious illnesses and their families. Improving palliative care knowledge among healthcare providers and the general population can lead to better support for patients during their end-of-life journey. This can be achieved through public education, social media initiatives, and the integration of palliative care content into the curriculum of medical science students especially medical and nursing students. In Iran, palliative care has not been incorporated into the medical education curriculum [63], and as a result, a large number of healthcare providers have no accurate information about what palliative care is and what its benefits are for patients [64]. Therefore, training programs for healthcare professionals on palliative care in heart failure patients could be developed and implemented to address this need. As a result, healthcare providers will be better equipped to provide compassionate and comprehensive care to those with incurable conditions, while the general population will gain a deeper appreciation for the importance of such care. Such efforts will ultimately contribute to a more compassionate and supportive healthcare system in Iran. Indeed, it is crucial for the government to prioritize and invest in designing and implementing culturally based palliative care programs. Supporting the development of these programs will ensure that patients with serious illnesses receive the necessary care and support during their end-of-life journey. By allocating resources and attention to palliative care initiatives, the government can improve the overall quality of healthcare services for those in need and their families. Moreover, investing in palliative care will lead to more compassionate and holistic care, enhancing the well-being and dignity of patients facing life-limiting conditions. Making palliative care a priority reflects the government's commitment to providing comprehensive and compassionate healthcare services to its citizens.

### Strengths and limitations

This was the first feasibility trial conducted in Iran, with the objective of integrating a palliative care approach into a nurse-led telehealth intervention for heart failure patients. Through this study, we successfully identified and recruited heart failure patients who experienced significant benefits from the early integrated palliative care interventions. Several limitations of this study are important to note. This was a single-site pilot feasibility study, which may limit the generalizability of the findings to other populations and settings. The short study duration and follow-up period may have limited the ability to detect significant changes in the secondary outcomes, so further research is needed to assess the long-term benefits of the intervention. As a feasibility pilot study, it was not adequately powered to detect a clinically significant difference in the secondary outcomes, which may have affected the ability to draw definitive conclusions from the results.

## Conclusions

To the best of our knowledge, this study is the first intervention that has been conceptualized, designed, and implemented to assess the feasibility and acceptability of a concurrent telehealth early palliative care intervention in Iran. The results showed that this intervention is feasible and acceptable for Iranian heart failure patients with NYHA Class II/ III HF. The results.

should inform the design of a definitive randomized controlled trials of this intervention for people with heart failure in Iran. The study's findings could encourage policymakers to invest in the development and implementation of similar interventions and to prioritize palliative care as an essential component of healthcare services for heart failure patients in Iran.

## Data Availability

All de-identified data are available upon reasonable request from corresponding author.

## Abbreviations

NYHA: New York Hospital Association
ACC: American College of Cardiology
ENABLE CHF-PC: Educate, Nurture, Advise, Before Life Ends Comprehensive Heartcare for Patients and Caregivers
HF: Heart Failure
IKHC: Imam Khomeini Hospital Complex
PKCCQ: Persian version of the Kansas City Cardiomyopathy Questionnaire
FACIT PAL-14: 14-Item Functional Assessment of Chronic Illness Therapy–Palliative-14 questionnaire
HADS: Hospital Anxiety and Depression Scale
ED: Emergencey Department
